# Acupuncture therapy in postoperative rehabilitation for arthroscopic rotator cuff repair: a systematic review and meta-analysis

**DOI:** 10.3389/fsurg.2025.1631880

**Published:** 2025-09-09

**Authors:** Jia-Hao Chen, Kai Wu, Qing-lai Wang

**Affiliations:** Wenzhou TCM Hospital Affiliated to Zhejiang Chinese Medical University, Wenzhou, China

**Keywords:** acupuncture therapy, arthroscopic rotator cuff repair, meta-analysis, postoperative rehabilitation, shoulder mobility

## Abstract

**Introduction:**

This study aimed to evaluate the efficacy of acupuncture therapy in the postoperative rehabilitation of patients following arthroscopic rotator cuff repair.

**Methods:**

Randomized controlled trials (RCTs) published up to October 31, 2024, were identified through systematic searches of EMBASE, Web of Science, China National Knowledge Infrastructure, China Science and Technology Journal Database, China Biomedical Literature Database, and Wanfang Database. Meta-analysis was conducted using RevMan 5.3 and Stata 18. A total of 18 studies involving 376 patients were included.

**Results:**

The meta-analysis demonstrated that, compared to the control group, the acupuncture group showed significantly higher effective rates [OR = 5.02, 95% CI (2.32, 10.85), *P* < 0.001], improved Constant-Murley scores [MD = 7.27, 95% CI (3.96, 10.59), *P* < 0.001], greater range of motion in forward flexion [MD = 14.95, 95% CI (6.13, 23.77), *P* < 0.001], backward extension [MD = 6.58, 95% CI (3.56, 9.61), *P* < 0.001], abduction [MD = 16.24, 95% CI (6.79, 25.70), *P* < 0.001], external rotation [MD = 9.56, 95% CI (4.66, 14.47), *P* < 0.001], and significantly reduced pain according to Visual Analog Scale scores [MD = −1.45, 95% CI (−2.04, −0.86), *P* < 0.001].

**Discussion:**

Acupuncture therapy, when compared to conventional treatments, may be beneficial for postoperative rehabilitation in patients after arthroscopic rotator cuff repair by reducing pain and improving shoulder joint mobility.

## 1 Introduction

Arthroscopic rotator cuff repair (ARCR) is the gold standard for treating rotator cuff injuries, with the advantages of less trauma, faster recovery and fewer complications (1). However, there are complications such as postoperative swelling and pain, shoulder stiffness, decreased muscle strength and muscle atrophy after ARCR (2, 3). Therefore, the availability of appropriate postoperative rehabilitation and the timing of rehabilitation are important factors in determining the degree of recovery of shoulder function in patients (4). At present, the main interventions are postoperative immobilization and functional exercises (5), but the lack of standardization of issues such as immobilization time and exercise modalities leads to poor rehabilitation outcomes (6, 7). Acupuncture is a therapeutic intervention within Traditional Chinese Medicine (TCM) that involves the insertion of fine metallic needles into specific anatomical points (acupoints) to regulate physiological functions or treat pathological conditions. According to the stimulation method, it is mainly categorized into hand acupuncture (e.g., millimeter acupuncture, wrist-ankle acupuncture, facial acupuncture, etc.), warm acupuncture and electroacupuncture. Relevant studies have showed its positive impact on interventions for shoulder pain symptoms (8–10). However, at present, a limited number of meta-analysis focus on acupuncture therapy for postoperative rehabilitation of ARCR. This paper systematically retrieved, organized, and screened randomized controlled trials (RCTs) focusing on the effects of acupuncture on postoperative rehabilitation of ARCR to prepare for a meta-analysis. The aim was to explore the potential benefits of acupuncture therapy in mitigating postoperative adverse effects associated with ARCR. By doing so, it sought to provide evidence-based support for the clinical application of acupuncture-assisted interventions in the postoperative rehabilitation of ARCR.

## 2 Data and methodology

### 2.1 Agreement and registration

This study was conducted in accordance with the PRISMA statement and AMSTAR guidelines (11, 12). The proposal reviewed is registered with PROSPERO (CRD42024604553).

### 2.2 Inclusion and exclusion criteria

#### 2.2.1 Inclusion criteria

(1) The type of study is a randomized controlled trial including the effect of hand acupuncture, warm acupuncture, electroacupuncture, and other acupuncture therapies on the rehabilitation of postoperative patients with ARCR, with unlimited languages; (2) Measures for the control group were blank, sham acupuncture, or conventional treatment, and measures for the intervention group were acupuncture or combined acupuncture therapy based on the control group; (3) At least one of the following outcome indicators was reported effective rate, visual analogue scores (VAS) (13), Constant-Murley scores (CMS) (14), and postoperative shoulder mobility (forward flexion, backward extension, shoulder abduction, and external rotation).

#### 2.2.2 Exclusion criteria

(1) Studies of patients who did not receive ARCR or other interventions included in the study; (2) Studies that the original text could not be obtained or the outcome indicators were not met; (3) Exclude non-RCT, duplicate publications, case reports, systematic reviews and meta-analysis, mechanism studies, dissertations, or animal experiments.

### 2.3 Document retrieval strategy

The computer search was conducted using EMBASE, Web of Science, China National Knowledge Infrastructure (CNKI), China Science and Technology Journal Database (CQVIP), China Biomedical Literature Database (SinoMed), and Wanfang Databases. This search was conducted to collect relevant clinical studies on the effects of acupuncture treatment on postoperative rehabilitation after ARCR from inception to October 31, 2024. The search method uses a combination of free words and subject terms, and is adapted to the different characteristics of each database. Table 1 provides the search strategy in detail.

**Table 1:** Detail of search strategy.

Database	Search details
EMBASE	(“rotator cuff repair”/exp OR “rotator cuff reconstruction”:ab,ti OR “shoulder cuff repair”:ab,ti OR “rotator cuff repair”:ab,ti) AND (“acupuncture”/exp OR “acupuncture therapy”:ab,ti OR “shonishin”:ab,ti OR “acupuncture”:ab,ti)
Web of Science	(((TS = (rotator cuff repair)) OR TS = (rotator cuff reconstruction)) OR TS = (shoulder cuff repair)) AND TS = (acupuncture)
CNKI	(篇关摘: 关节镜下肩袖修补术后 + 肩袖修补术后 + 肩袖损伤(精确))AND篇关摘: 针刺疗法 + 电针 + 浮针 +温针(精确))AND(篇关摘:随机对照 + 随机 + RCT(精确))
CQVIP	篇关摘=针 + 腕踝针 + 揿针 + 耳穴 + 电针 + 浮针 + 针灸 + 针疗法 + 肩三针 AND 篇关摘=肩袖修补术 + 关节镜下肩袖修补 + 肩袖损伤术后
SinoMed	(“肩袖修补术”[常用字段:智能] OR “关节镜下肩袖修补”[常用字段:智能] OR “肩袖损伤术后”[常用字段:智能]) AND(“针”[常用字段:智能] OR “腕踝针”[常用字段:智能] OR “揿针”[常用字段:智能] OR “耳穴”[常用字段:智能] OR “电针”[常用字段:智能] OR “浮针”[常用字段:智能] OR “针灸”[常用字段:智能] OR “针疗法”[常用字段:智能] OR “肩三针”[常用字段:智能])
Wanfang	(全部:(关节镜下肩袖修补术后 or 肩袖修补术 or 肩袖损伤 or 肩袖) and 全部:(针刺疗法 or 针刺 or 针疗法 or 浮针 or 穴位刺激) and 全部:(随机对照 or 对照 or RCT)) Word variations have been searched

### 2.4 Document screening and data extraction

The literature was managed using EndNote 21.4 software to summarize, de-duplicate, screen and extract research data. Initial screening was carried out by 2 independent researchers (Jia-Hao Chen and Wu Kai) using titles and abstracts, followed by re-screening using full text. In case of disagreement, it shall be resolved through discussion or consultation with the corresponding author (Qing-Lai Wang). The content of data extraction mainly includes author names, year of publication, sample size, interventions, and outcome indicators, etc.

### 2.5 Include research quality assessment

The Cochrane Collaboration Tool was used to assess the risk of bias of the included literature that met the inclusion criteria. The risk of bias was assessed as “low risk”, “high risk”, “unclear”. Disagreements were decided by discussion or by requesting the correspondent author.

### 2.6 Statistical analysis

Statistical analysis was conducted using RevMan 5.4 and Stata 18 statistical software. The odds ratio (OR) and its 95% confidence interval (CI) were used for dichotomous variables, and the mean difference (MD) and its 95% CI were used for continuous variables. The *I²* test was used to assess heterogeneity between research results. When the statistical heterogeneity was low (*P* > 0.1, *I*^2^ < 50%), the fixed-effects model was used for analysis. When statistical heterogeneity was significant (*P* < 0.1, *I*^2^ > 50%), a random-effects model was used, and one-way sensitivity analysis was performed to assess the effect of included studies on the combined results for outcomes with significant heterogeneity. In cases where a unified effect value is not available for outcome indicators, a weighted mean ($\bar{X}$) and combined standard deviation (S) is calculated using the formula below:

$$\bar{X}=\frac{\sum_{i=1}^{n}\;({\bar{x}}_{i}\;\cdot \;n_{i})}{\sum_{i=1}^{n}\;n_{i}}$$

$$S=\sqrt{\frac{\sum_{i=1}^{n}\;((n_{i}-1)S_{i}^{2}+n_{i}{\left({\bar{x}}_{i}-\bar{x}\right)}^{2})}{\sum_{i=1}^{n}\;n_{i}-1}}$$

here ${\bar{x}}_{i}$ denotes mean, $S_{i}$ denotes standard deviation, $n_{i}$ denotes sample size. For indicators with ≥10 included studies, meta-regression analysis was performed using Stata 18.0 software to explore sources of heterogeneity, and funnel plots, Egg tests, and Begg tests were constructed to assess publication bias (15). *P* < 0.05 was considered statistically significant publication bias. Evidence was graded using the GRADEpro software.

## 3 Result

### 3.1 Literature search results and bias risk assessment included in the study

**Figure 1:**
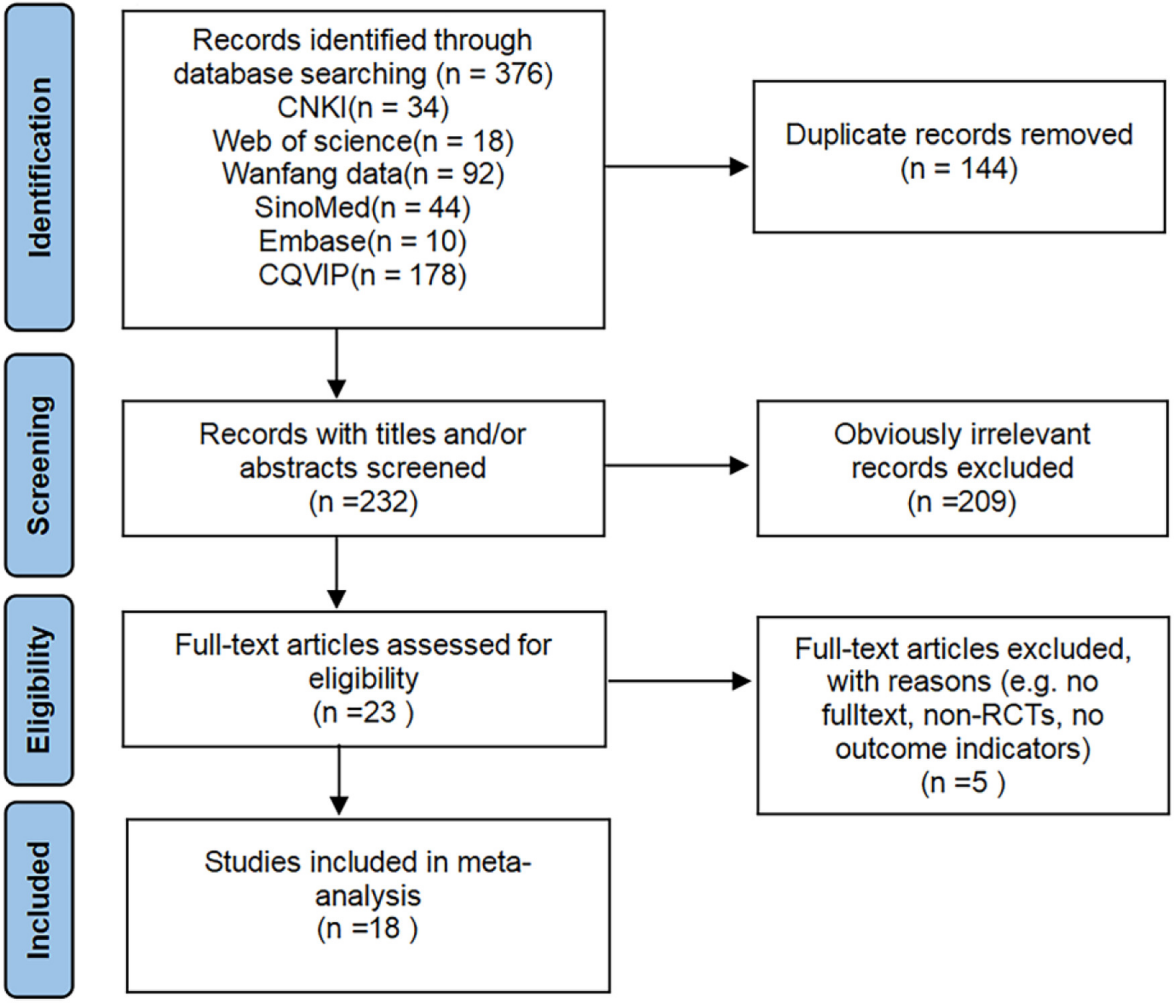
Literature screening flow diagram.

**Figure 2:**
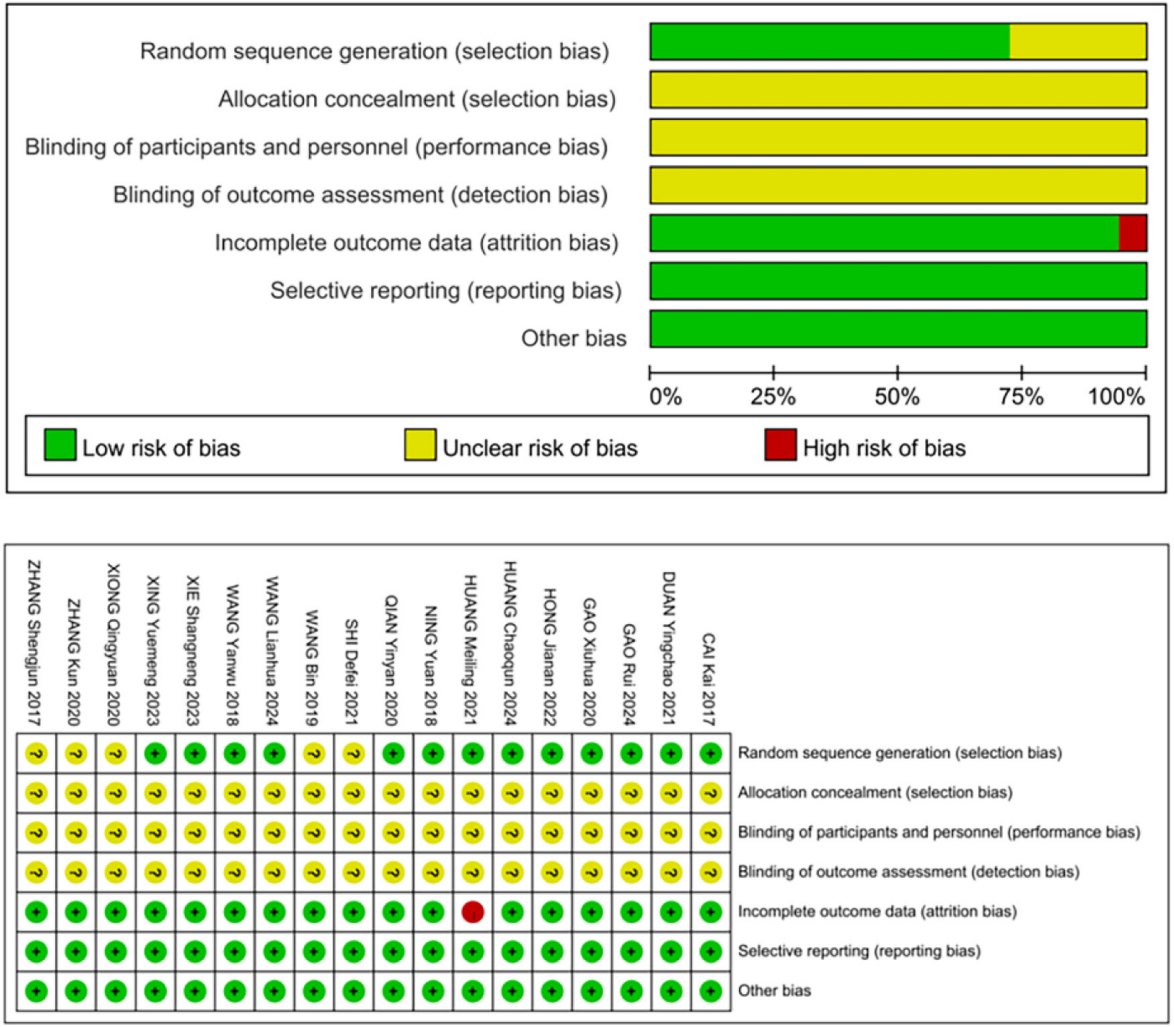
Bias risk assessment chart.

A total of 376 related literatures were initially retrieved, of which 144 were omitted due to repetition. Finally, 18 studies (16–33) of RCTs were included after excluding 214 studies based on title, abstract and full text. The publication years ranged from 2017 to 2024, with a total of 1304 patients, of which 652 were in the control group and 652 were in the acupuncture group. The literature screening process is shown in Figure 1. The basic characteristics of the included studies are shown in Table 2. All references refer to randomization, of which 13 studies (16–21, 23–26, 30, 31, 33) use the random number table method and 5 studies (22, 27–29, 32) refer to randomization without describing the method of random sequence generation. The quality assessment of the included studies is shown in Figure 2, and the result shows that the overall quality was good.

**Table 2:** Characteristics of the included studies.

Study, Year [Reference]	Sample size		Intervention			Outcome indicators
	Acu	Control	Acu	Control	Acupoints selection	
Gao and Zhong (16)	30	30	Electro	Routine	LI14, LI15, etc	②
Huang et al. (17)	62	62	Facial + Prednisone	Prednisone	Shoulder poin, Elbow point, Upper Burner	③
Wang et al. (18)	46	46	Wrist-ankle	Routine	Upper 4, 5, 6 zone of the affected side	②④
Xie et al. (19)	30	30	Electro + Myoelectricity	Myoelectricity	SI9, LI14, LI15, GB21	②④
Xing et al. (20)	35	37	Wrist-ankle	Routine	Upper 4, 5, 6 zone of the affected side	②④
Hong and Lei (21)	35	35	Millimetre	Routine	BL64,SI9,SI3,SJ14,LI15, etc	①②
Shi (22)	33	32	Warm	Routine	LI14, LI15, SI9	①③
Duan et al. (23)	46	46	Electro	Routine	LI15, LI14, SJ14	①②③④
Huang et al. (24)	30	30	Warm	Routine	LI15, SI9, LI14	②
Gao et al. (25)	32	32	Superficial	Routine	Myofascial lines	②
Qian and Shou (26)	40	40	Wrist-ankle	Routine	Upper 4, 5 zone of the affected side	②③
Xiong et al. (27)	30	30	Wrist-ankle + Dizocin	Dizocin	Upper 2, 4, 5 zone of the affected side	②③
Zhang (28)	38	37	Millimetre	Routine	SI9, SJ14, LI15	①②④
Wang and Liu (29)	30	30	Millimetre	Routine	LI15, SJ14, LI14, GB21	②
Ning et al. (30)	30	30	Electro	Routine	LI15, LI14, SJ14	②③
Wang et al. (31)	40	40	Electro	Routine	SI9, SJ14, LI15, SI11, LI14	②④
Zhang et al. (32)	35	35	Warm	Routine	LI14, LI15, SI9	③
Cai (33)	30	30	Warm	Routine	LI14, LI15, SI9	②③

①: effective rate; ②: VAS scores; ③: CMS scores; ④: shoulder mobility; Acupoints selection is according to the WHO international standard terminologies.

### 3.2 Meta analysis results

#### 3.2.1 Effective rate

**Figure 3:**
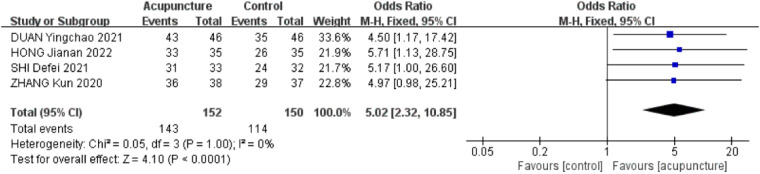
Forest plot of effective rate.

A total of 4 RCTs (21–23, 28) were included. The heterogeneity between the included research results was low (*I*^2^ = 0%, *P* = 1.00), so a fixed-effects model was used for meta-analysis. The results showed that the effective rate in the acupuncture group was significantly higher than that in the control group, and the difference was statistically significant [OR = 5.02,95% CI (2.32,10.85), *P* < 0.001, Figure 3].

#### 3.2.2 VAS scores

**Figure 4:**
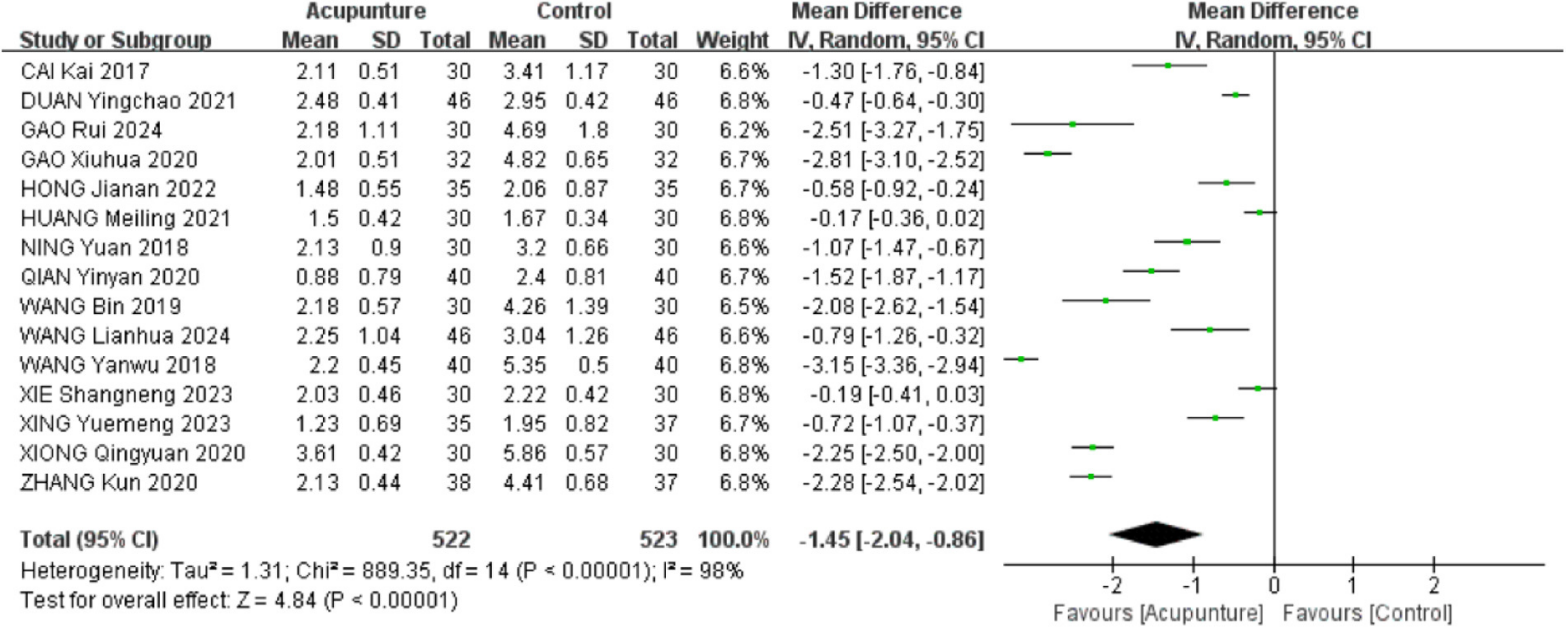
Forest plot of VAS scores.

A total of 15 RCTs (16, 18–21, 23–31, 33) were included. The heterogeneity of the included research results was high (*I*^2^ = 98%, *P* < 0.001), so a random effects model was used for meta-analysis. The results showed that the VAS scores in the acupuncture group were significantly lower than that in the control group, and the difference was statistically significant [MD = −1.45, 95% CI (−2.04, −0.86), *P* < 0.001, Figure 4]. A meta-regression analysis was conducted using acupuncture type, treatment duration, and concurrent rehabilitation training as covariates. The findings indicated that acupuncture type (*β* = −.23, *P* = .256), treatment duration (*β* = −.20, *P* = .279), and concurrent rehabilitation training (*β* = .43, *P* = .589) did not exhibit statistical significance on the outcome, with small effect sizes. The adjusted *R*^2^ of 5.01% suggests that these variables offer limited explanation for inter-study heterogeneity, indicating other potential confounders such as stimulation intensity and acupoint selection.

#### 3.2.3 CMS scores

**Figure 5:**
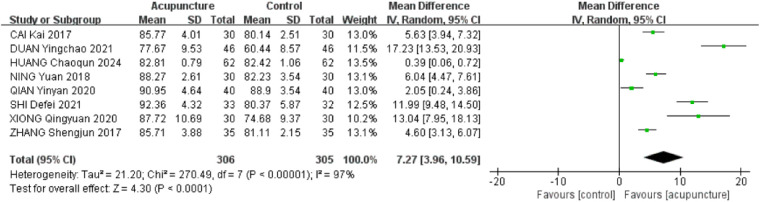
Forest plot of CMS scores.

A total of 8 RCTs (17, 22, 23, 26, 27, 30, 32, 33) were included. The heterogeneity of the included research results was high (*I*^2^ = 97%, *P* < 0.001), so a random-effects model was used for meta-analysis. The results showed that the CMS scores in the acupuncture group was significantly higher than that in the control group, and the difference was statistically significant [MD = 7.27, 95% CI (3.96,10.59), *P* < 0.001, Figure 5].

#### 3.2.4 Shoulder mobility in forward flexion

**Figure 6:**
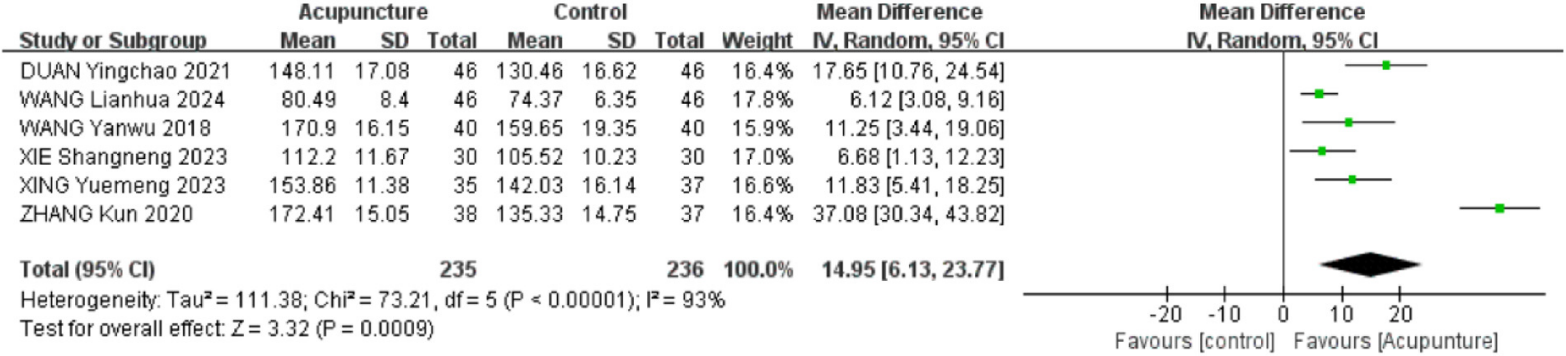
Forest plot of shoulder mobility in forward flexion.

Six RCTs (18–20, 23, 28, 31) were included. The heterogeneity of the included research results was high (*I*^2^ = 93%, *P* < 0.001), so a random effects model was used for meta-analysis. The results showed that the acupuncture group had a significant increase in forward flexion compared with the control group, and the difference was statistically significant [MD = 14.95, 95% CI (6.13,23.77), *P* < 0.001, Figure 6].

#### 3.2.5 Shoulder mobility in backward extension

**Figure 7:**
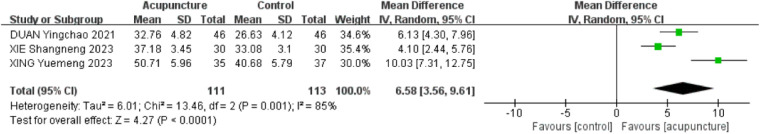
Forest plot of shoulder mobility in backward extension.

Three RCTs (19, 20, 23) were included. The heterogeneity of the included research results was high (*I*^2^ = 85%, *P* = 0.001), so a random effects model was used for meta-analysis. The results showed that the acupuncture group had a significant increase in backward extension compared to the control group, and the difference was statistically significant [MD = 6.58, 95% CI (3.56,9.61), *P* < 0.001, Figure 7].

#### 3.2.6 Shoulder mobility in abduction

**Figure 8:**
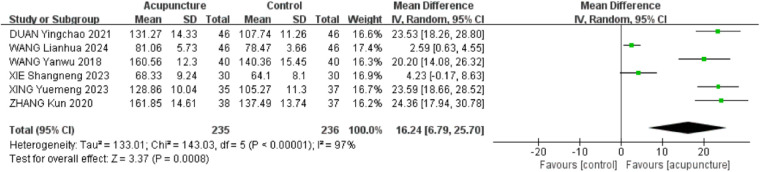
Forest plot of shoulder mobility in abduction.

Six RCTs (18–20, 23, 28, 31) were included. The heterogeneity of the included research results was high (*I*^2^ = 97%, *P* < 0.001), so a random effects model was used for meta-analysis. The results showed that the acupuncture group had a significant increase in abduction compared to the control group, and the difference was statistically significant [MD = 16.24, 95% CI (6.79,25.70), *P* < 0.001, Figure 8].

#### 3.2.7 Shoulder mobility in external rotation

**Figure 9:**
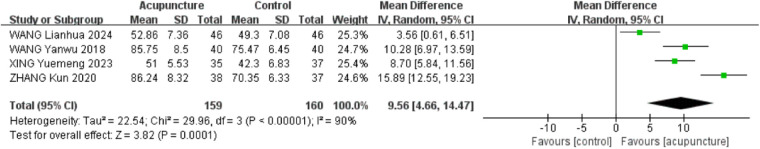
Forest plot of shoulder mobility in external rotation.

Four RCTs (18, 20, 28, 31) were included. The heterogeneity of the included research results was high (*I*^2^ = 90%, *P* < 0.001), so a random effects model was used for meta-analysis. The results showed that the acupuncture group had a significant increase in external rotation compared to the control group, and the difference was statistically significant [MD = 9.56, 95% CI (4.66,14.47), *P* < 0.001, Figure 9].

#### 3.2.8 Adverse reactions

**Figure 10:**
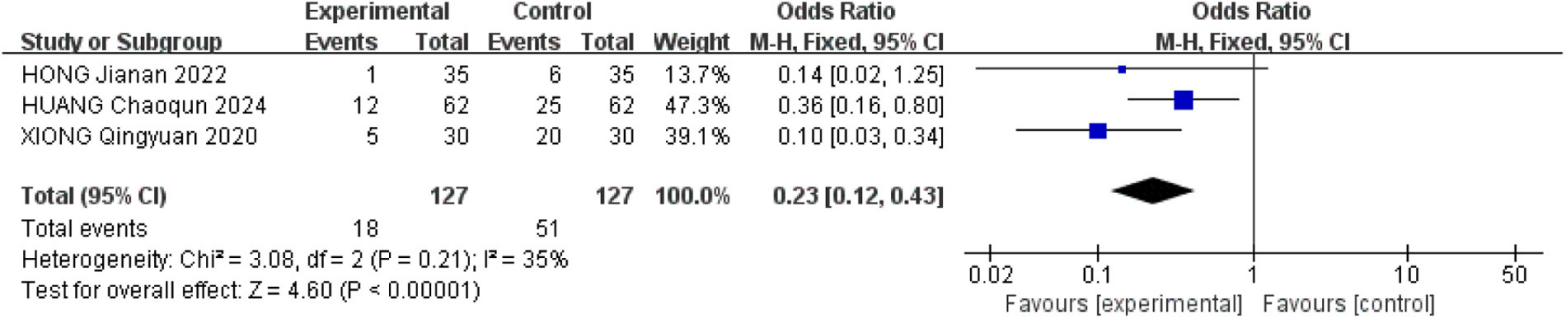
Forest plot of adverse reactions.

Adverse reactions were assessed in eighteen studies included in the analysis. Among these, eleven studies (16, 19, 22, 25, 26, 28–30, 32, 33) did not provide safety information, while seven studies (17, 18, 20, 21, 24, 27, 31) reported on adverse reactions. Specifically, four studies (18, 20, 24, 31) explicitly stated the absence of adverse reactions, while three studies (17, 21, 27) detailed a total of 66 adverse reactions. Moderate heterogeneity (*P* = 0.23, *I*^2^ = 35%) was observed among the study populations, leading to the utilization of a fixed-effects model. The analysis revealed a significant reduction in the risk of adverse reactions in the treatment group (OR = 0.23, 95% CI: 0.21–0.43, *P* < 0.001), indicating a statistically significant difference. The predominant adverse events reported were dizziness, pain, and mild gastrointestinal symptoms, supporting the favorable safety profile of acupuncture therapy for ARCR. These findings are represented in Figure 10.

### 3.3 Sensitivity analysis

**Figure 11:**
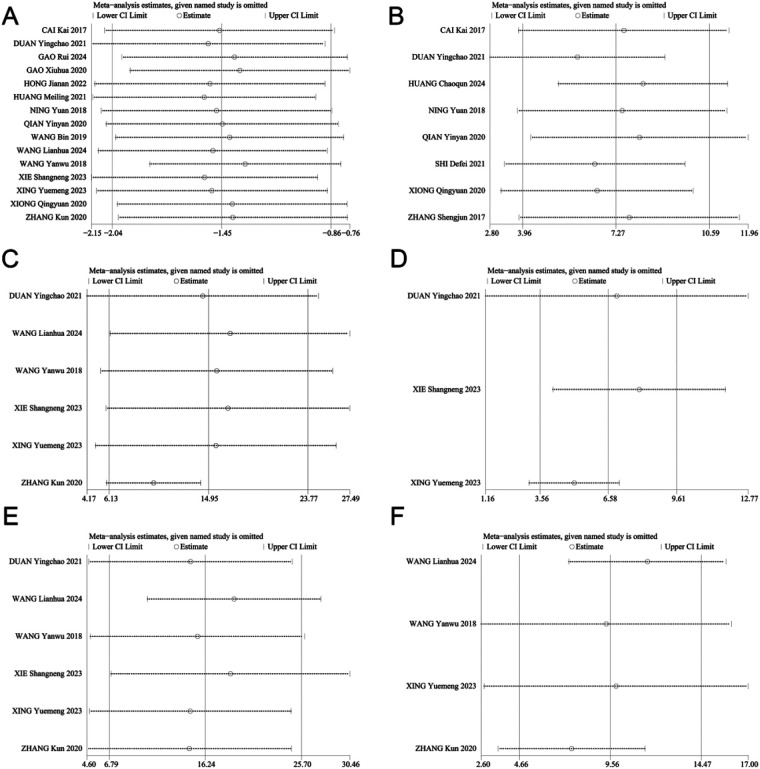
Sensitivity analysis of **(A)** VAS scores, **(B)** CMS scores, **(C)** forward flexion, **(D)** backward extension, **(E)** shoulder abduction, **(F)** external rotation.

The heterogeneity among the VAS scores, CMS scores, and shoulder mobility was substantial. Therefore, sensitivity analysis was performed to determine the source of heterogeneity for these outcomes. The sensitivity analysis was shown in Figure 11. The results remained consistent, suggesting that the stability of this study is good and the results are reliable.

### 3.4 Analysis of publication bias

**Figure 12:**
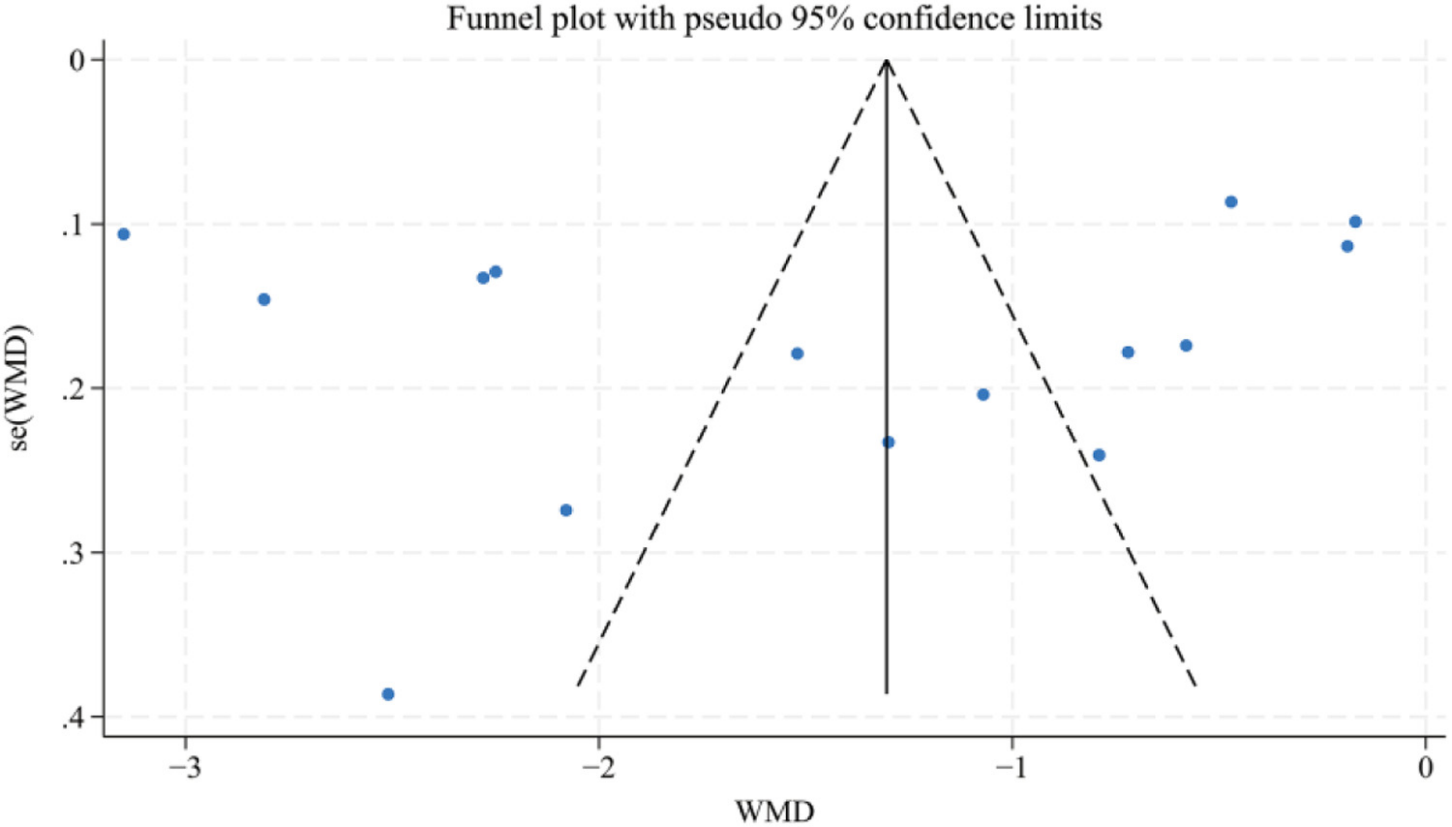
Funnel plot for VAS scores.

**Figure 13:**
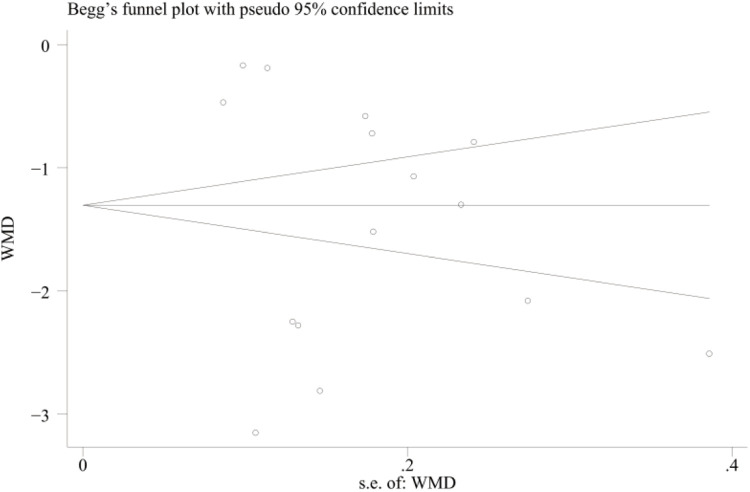
Begg's publication bias plot for VAS scores.

**Figure 14:**
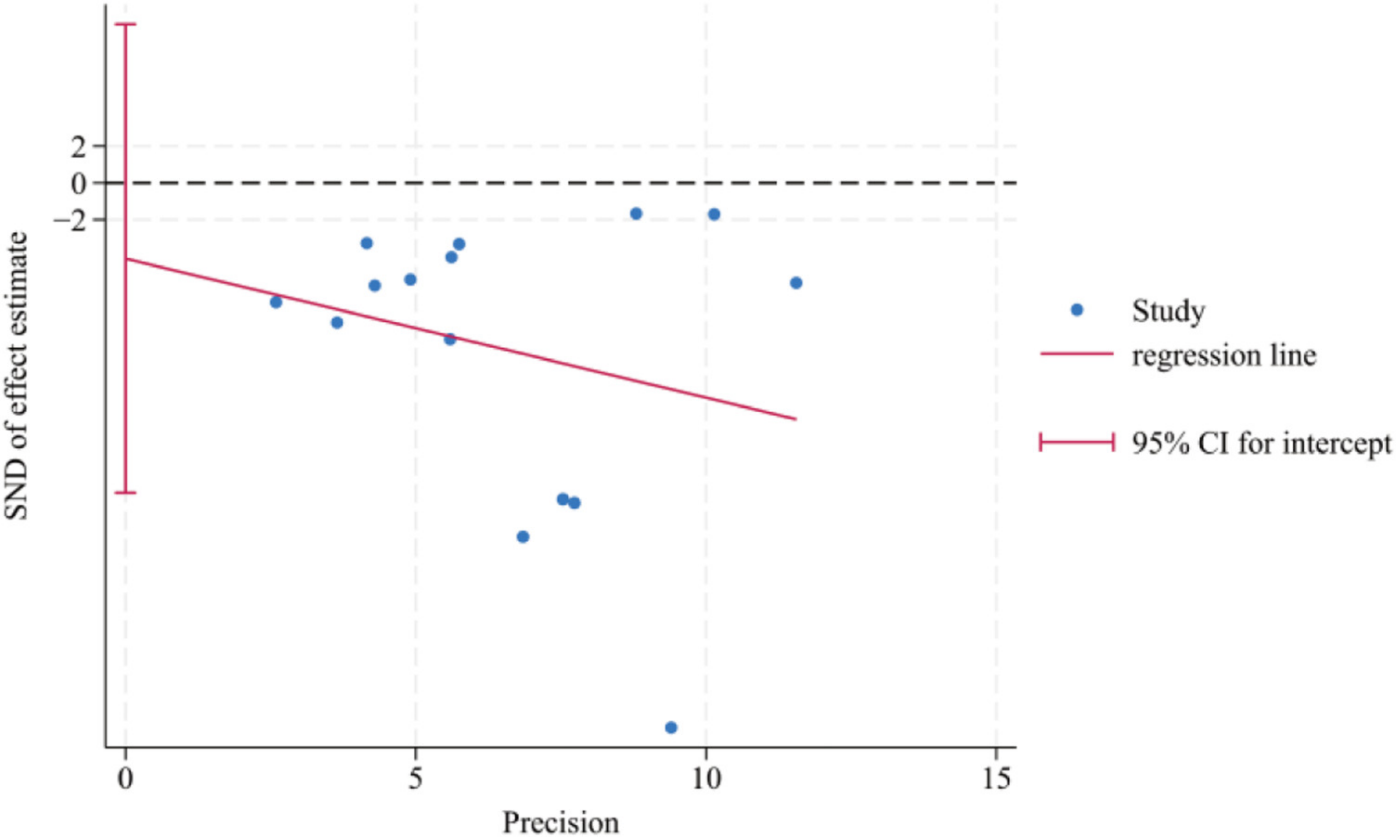
Egger's publication bias plot for VAS scores.

The VAS scores (*n* > 10) were analyzed using the Egger's test and the Begg's test for publication bias, and the results showed that there was no publication bias (Egger's test, *t* = −0.70, *P* = 0.497 > 0.05), (Begg's test, *P* = 0.322 > 0.05). In addition, the funnel plot showed that the distribution of each study point was basically symmetrical, indicating that there was no publication bias (Figures 12–14). Although there was a small amount of bias, the overall results were acceptable.

### 3.5 GRADE assessment of evidence quality

**Figure 15:**
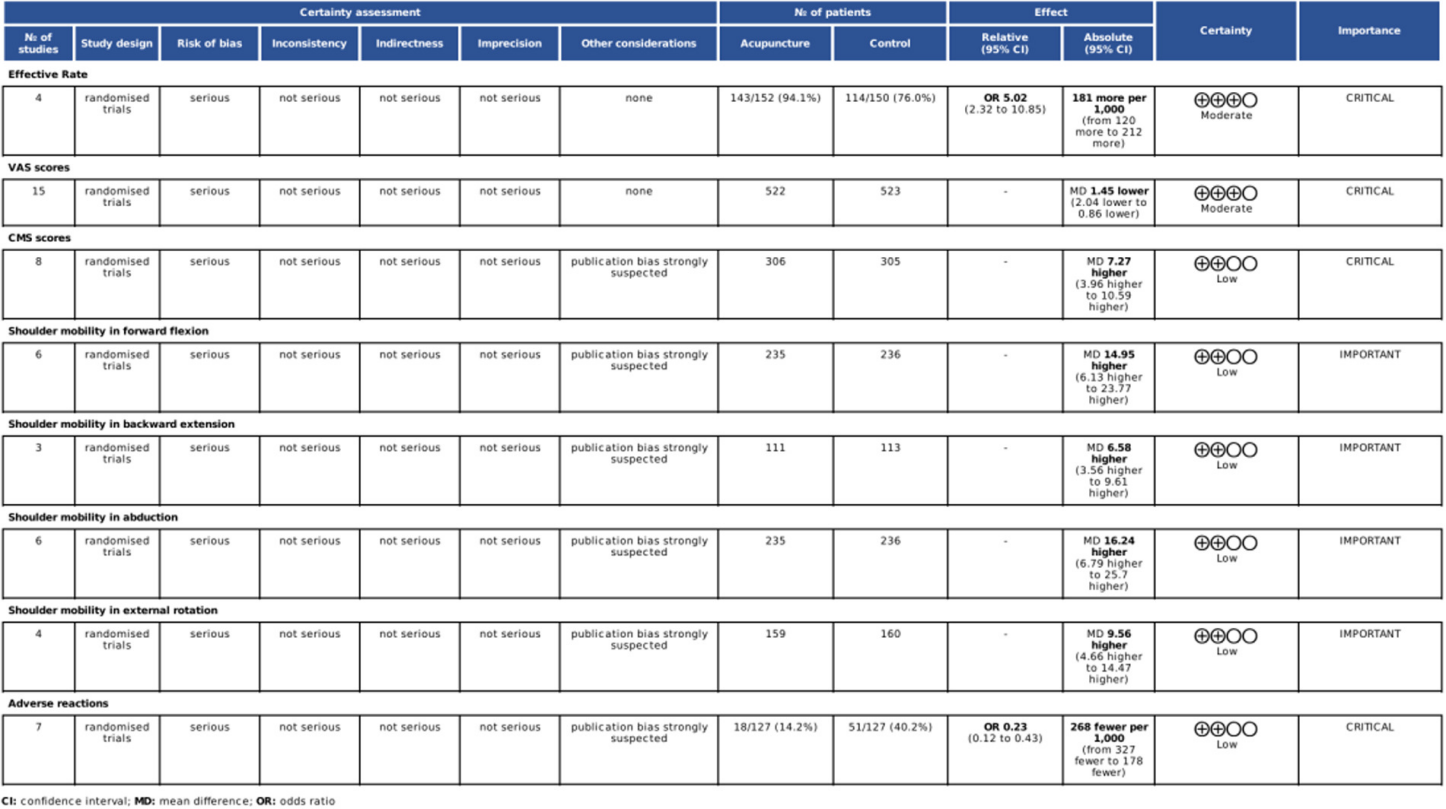
GRADE assessment of evidence quality.

The GRADE assessment of evidence quality revealed that the effective rate and VAS scores were supported by moderate-quality evidence, while the CMS scores, shoulder mobility and adverse reactions were supported by low-quality evidence. Evidence degradation was primarily attributed to factors such as inadequate distribution concealment, absence of blinding, publication bias, and significant heterogeneity among studies, all of which undermine the reliability of the research findings. The results are shown in Figure 15.

## 4 Discussion

This meta-analysis aimed to quantify the impact of acupuncture therapy on postoperative rehabilitation for ARCR by summarizing the current RCTs results. It was found that acupuncture therapy showed a significant improvement in effective rate (OR = 5.02), VAS scores (MD = −1.45), CMS scores (MD = 7.27) and shoulder mobility in forward flexion (MD = 14.95), backward extension (MD = 6.58), shoulder abduction (MD = 16.24), external rotation (MD = 9.56). At present, few meta-analysis focus on acupuncture therapy intervention in postoperative rehabilitation for ARCR. This study aims to contribute to this under-explored area. Our conclusions provide some evidence-based support for adjunctive intervention of acupuncture therapy in postoperative rehabilitation of ARCR and confirm its potential therapeutic benefits. Despite heterogeneity among the studies included in the paper, publication bias detection and sensitivity analysis were performed separately, which indicated stable and reliable results.

The selected acupoints (LI14, LI15, SJ14) were chosen based on the Traditional Chinese Medicine (TCM) principle of “Local Points Selection” (34). From the TCM perspective, stimulating these specific points along the Hand Yangming Large Intestine meridian can promote meridian flow and regulate qi, which is considered beneficial for treating shoulder disorders (35). From a biomedical perspective, modern research has showed that acupuncture at these points can stimulate peripheral nerves and activate specific brain regions. For instance, LI14 and LI15 correspond to areas innervated by branches of the brachial plexus, particularly the axillary nerve and radial nerve. Acupuncture stimulation in these regions may help modulate pain transmission through the gate-control mechanism and activate endogenous pain-modulatory systems (36). Besides, functional MRI studies have shown that acupuncture at specific points can activate brain regions involved in pain processing and motor control, including the primary somatosensory cortex and motor cortex (37). Furthermore, these acupoints also show significant overlap with trigger points identified in myofascial pain syndrome, suggesting their potential role in releasing muscle tension and improving local circulation (38). Thus, The combination of LI14, LI15, and SI9, known as the “three shoulder points” in TCM, corresponds with Western medicine's understanding of comprehensive neuromuscular modulation in treating soft tissue shoulder disorders.

The effect of acupuncture on postoperative ARCR may be related to the following mechanisms: First, according to TCM theory, acupuncture is believed to facilitate the movement of qi and blood, and regulate the body's yin and yang balance. This corresponds to pain relief and the alleviation of soft tissue spasms in modern medicine (39, 40). Second, electroacupuncture can broaden the scope of acupoint stimulation by incorporating neuromuscular electrical stimulation, which helps to inhibit hyperreflexia and nociceptive hypersensitivity in the damaged area. Furthermore, it promotes the regeneration and repair of peripheral neuron axons, facilitating reinnervation. This process subsequently improves the function of innervated organs and prevents muscle atrophy (41–43). Finally, the heat conduction of warm acupuncture is transmitted to the acupoints through the handle of the needles along the needle. This process has been shown to enhance the induction of thermal stimulation at acupoints, promote the absorption of local inflammatory factors, thereby alleviating the pain caused by inflammatory factors in the shoulder joint. Additionally, it facilitates the expansion of blood vessels and lymphatic vessels in shoulder lesions, improving blood circulation, which in turn reduces inflammatory exudation and mitigates the adhesion of shoulder soft tissues (44–46).

Our research may have some limitations. Firstly, this paper included studies involving different types of acupuncture, acupoints and stimulus intensity, which may cause different outcome measures and generate heterogeneity. Secondly, all studies failed to mention blinding. and some of the literature had missed visits, which may cause selection bias and affect the reliability of the study results. Moreover, ARCR includes both small incision rotator cuff repair and total arthroscopic rotator cuff repair. However, this study did not restrict the type of surgery, which may have overlooked the impact of the surgery type on postoperative pain and joint mobility.

## 5 Conclusion

This study shows that acupuncture therapy may be beneficial for postoperative rehabilitation in patients after ARCR, as evidenced by improvements in the effective rate, CMS scores, and shoulder motion function, along with a notable reduction in VAS scores. The results must be viewed with caution due to the preliminary and heterogeneous nature of the included evidence. In the future, more well-designed, large sample size, high quality studies are needed to further validate the scientific validity of the conclusions.

## Data Availability

The original contributions presented in the study are included in the article/Supplementary Material, further inquiries can be directed to the corresponding author.
